# Malnutrition in elder care: qualitative analysis of ethical perceptions of politicians and civil servants

**DOI:** 10.1186/1472-6939-11-11

**Published:** 2010-06-16

**Authors:** Anna-Greta Mamhidir, Mona Kihlgren, Venke Soerlie

**Affiliations:** 1 Department of Neurobiology, Caring Sciences and Society, Karolinska Institute, Stockholm, Sweden; 2 Department of Health and Caring Sciences, University of Gävle, Sweden; 3 Centre for Nursing Science, Örebro University Hospital, Örebro, Sweden; 4 Bodo University College, School of professional studies, Centre for Practical Knowledge, Norway

## Abstract

**Background:**

Few studies have paid attention to ethical responsibility related to malnutrition in elder care. The aim was to illuminate whether politicians and civil servants reason about malnutrition in elder care in relation to ethical responsibility, and further about possible causes and how to address them.

**Method:**

Eighteen elected politicians and appointed civil servants at the municipality and county council level from two counties in Sweden were interviewed. They worked at a planning, control and executive level, with responsibility for both the elder care budget and quality of care. Qualitative method was used for the data analysis.

**Results:**

Two themes emerged from their reasoning about malnutrition related to ethical responsibility. The theme *assumed role *involves the subthemes *quality of care and costs, competent staff and govern at a distance*. Old and ill patients were mentioned as being at risk for malnutrition. Caregivers were expected to be knowledgeable and stated primary responsible for providing adequate nutritional care. Extended physician responsibility was requested owing to patients' illnesses. Little was reported on the local management's role or on their own follow-up routines. The theme *moral perception *includes the subthemes *discomfort, trust and distrust*. Feelings of discomfort concerned caregivers having to work in a hurried, task-oriented manner. Trust meant that they believed for the most part that caregivers had the competence to deal appropriately with nutritional care, but they felt distrust when nutritional problems reappeared on their agenda. No differences could be seen between the politicians and civil servants.

**Conclusion:**

New knowledge about malnutrition in elder care related to ethical responsibility was illuminated by persons holding top positions. Malnutrition was stressed as an important dimension of the elder care quality. Governing at a distance meant having trust in the staff, on the one hand, and discomfort and distrust when confronted with reports of malnutrition, on the other. Distrust was directed at caregivers, because despite the fact that education had been provided, problems reappeared. Discomfort was felt when confronted with examples of poor nutritional care and indicates that the participants experienced failure in their ethical responsibility because the quality of nutritional care was at risk.

## Background

The problematic issue of malnutrition in elder care is regularly reported on [1-3], but few studies have paid attention to ethical responsibility in relation to malnutrition. In a public healthcare system, the overall responsibility for the budget and quality of elder care rests with governmental politicians and civil servants, i.e. with those in high positions and with power. Politicians and civil servants are representatives of the public and the professionals, which mean that they have to safeguard the existence of ethically defensible elder care [4]. All professionals are responsible for the care they provide, and the code of ethics for nurses [5] emphasizes their ethical responsibility to protect frail patient groups. Among older and ill patients, deteriorated overall health [6] and decreased well-being are some of the serious consequences of malnutrition [7].

In the healthcare system, ethical problems related to responsibility have been illuminated among different professionals, and in different contexts and situations [8-10]. The ethically challenging situations confronting politicians and civil servants responsible for elder care have been illuminated [11]. They have been shown to experience ethical conflicts associated with lack of good care, such as when vulnerable patients are placed in inappropriate care settings [11].

Caring for and caring about others demonstrates responsibility and trust [12]. Trust is fundamental to human beings and not only of concern to individuals, but also to institutions, and for this reason attention needs to be paid to institutional structures that are in a position to cause harm [13].

The causes of malnutrition are multi-factorial, where chronic illness [14,15], cognitive and physical impairments [16], depression, loss of appetite [17,18], stroke, eating dependencies [19], problems with chewing [20] and swallowing difficulties [21] are among the most important risk factors.

Malnutrition should be viewed as an aspect of quality care associated with the healthcare system [4]. Improper nursing care related to nutritional issues should be discussed in light of the codes of ethics for nurses [22], because if staff is not properly supervised and if they are overburdened, the quality of the care they provide will be compromised.

The development of malnutrition may be slowed, prevented or reversed if it is identified [23]. Mattsson-Sydner [24] described how lack of communication and cooperation in the healthcare system between the different levels involved in and responsible for nutrition could result in a sense of powerlessness and feelings of non-accountability. Caregivers are responsible for providing adequate nutrition on a daily basis. Politicians' and civil servants' decisions and work affect many stakeholders: the patients, their relatives and the various healthcare providers involved. Few studies have focused on persons holding top positions and their reasoning about nutritional issues and malnutrition in elder care. Therefore, it is important to get a deeper understanding of their thoughts and experiences regarding these issues. The aim was to illuminate whether politicians and civil servants reason about malnutrition in elder care in relation to ethical responsibility, and further about possible causes and how to address them.

## Method

### **Participants, setting and procedure**

The sample consisted of eighteen elected politicians and appointed civil servants at the municipality and county council level from two counties in Sweden. Inclusion criteria for the participants were; holding top positions at a planning and control as well as executive level, and had responsibility for both the elder care budget and quality of care. Additionally, at least one year of experience in the position was requested. The names of the politicians and civil servants were compiled on a list from the two counties. The sampling procedure consisted of drawing names from the list until the desired number of participants was achieved. Thereafter the participants were contacted by telephone, information about the aim of the study, the procedure as well as written information was provided. Confidentiality was assured, and the design ensured no possibility of tracing the findings to the participants. Written consent was given after both verbal and written information had been provided. The Regional Research Ethical Committees approved this study (99310-17). All eighteen individuals agreed to participate, and date and location for the interviews were decided. The participants were between 43 to 66 years of age, 13 were female and they had held their positions from one to 20 years.

### Data collection

#### Interviews

Interviews were used for the data collection in this study. The interviews were a part of a larger project focusing on the meaning of being in ethically difficult situations related to elder care as experienced by politicians and civil servants [4]. The present interviews started after a pause for about 15 minutes. Before the start, the interviewer highlighted the reports and the research results of problematic nutritional issues and malnutrition in elder care. This was meant to support the participants in their reflections of the area of concern. An open ended question was addressed and they were asked '*Please tell me about your experiences and perceptions of malnutrition in elder care and about the ethical responsibility'*. The politicians and civil servants were encouraged to present their reflections without interruption so that the narrative contents would be as rich as possible. Follow-up questions were asked when the interviewer wanted them to elaborate further or provide a clarification. Follow-up questions concerned the participants' thoughts, feelings and actions [25]. The interviews were tape-recorded and transcribed verbatim and took between 25-40 minutes to perform. Notes were taken during the interviews to help with orientation and understanding in the analysis phase and included non-verbal communication. The politicians and civil servants were chose the location for the interviews, which was often their office.

### Data analysis

#### Interpretations

A phenomenological hermeneutic method [25] was used for the data analysis. This method has been used by other authors [9,11] and is useful when attempting to elucidate the meaning of a lived experience through interpretation of an individual's narrative. The phenomenological hermeneutic analysis process consists of three phases: the *naïve reading*, one or more *structural analyses *and a *comprehensive understanding*. The analysis process constitutes a dialectal movement between the whole and the parts of the text and between understanding and explanation [26].

In the first phase, a *naïve reading *of the transcribed interviews was carried out to obtain a first impression of the text as a whole concerning the politicians' and civil servants' reasoning and experiences with regard to malnutrition in elder care in relation to responsibility. The naïve reading indicated the direction for subsequent analyses. The narratives were shown to include both illumination of moral perceptions and assumed obligations. In the second phase, *structural analyses *were performed, which are detailed analyses of the text conducted to explain the parts and validate or invalidate the initial understanding gained through the naïve reading. The text was divided into meaning units that were then condensed, abstracted and structured into subthemes and themes [25]. A meaning unit can be part of a sentence, a whole sentence or a paragraph. The subthemes and themes are presented under the heading Results. A *comprehensive understanding *was developed in the third phase, where the authors' pre-understandings, the naïve reading, the structure analyses and relevant literature were taken into account. This is addressed under the heading Discussion. All authors took part in the analyses until agreement on the interpretation and findings were considered satisfactory.

## Results

The naïve reading revealed two different aspects of the narratives: the first concerned the assumed role in the healthcare system and the second the moral perceptions. The theme *assumed role *includes the subthemes *quality of care and costs, competent staff and govern at a distance*. The theme *moral perception includes the subthemes discomfort, trust and distrust*. Quotations from the interviews are presented. No differences could be seen between the politicians and the civil servants or between the municipality and county council levels regarding this issue.

### Assumed role

#### Quality of care and costs

The politicians and civil servants reported that adequate nutrition is an important dimension of quality in elder care. They spoke of their responsibility for the budget and quality of care owing to their assumed role and mandate, which meant that they had an obligation both to control costs and to ensure the quality of care. Malnutrition among older ill patients was mentioned as only one of several problematic fields that needed to be dealt with. The participants experienced difficulties achieving quality in elder care, particularly in relation to the simultaneous staff reductions and increased needs of elder care. They stated that, in their decision-making and role regarding elder care, they had several goals and directions to take into consideration, not only the area of nutrition.

'To eat and have good meals, that is important for everyone, and of course for the older patients'

'The budget you know, personnel reductions due to budget cuts are a reality and at the same time patient care needs have increased, this is true, it is difficult'

Another aspect of quality of care mentioned by the politicians and civil servants was that they believed it was difficult to reverse the conditions of malnutrition for some of the ill patients. They reflected on the patients' great age, often several illnesses, dependency on others for food intake and cognitive impairment. The normal decline in health status found with advanced age was a risk factor, according to them. They felt these patients were very vulnerable.

'They are old and sick, malnutrition is to be expected and there's probably nothing much that can be done, they are really vulnerable'

#### Competent staff

The politicians and civil servants considered that caregivers have the primary responsible for ensuring patients' nutritional status. They expected caregivers to have functioning routines, and to notice and be able to identify nutritional problems because they are so close to the patients. They stated that caregivers had to increase their awareness of nutritional issues as well as their understanding of the different aspects involved. Furthermore, physicians' knowledge should be used to promote nutritional care owing to patients' poor health status.

'The caregivers are responsible for the patients, their eating, it's their job'

'The patients are old and sick, therefore the physicians need to be involved'

They expected caregivers to be knowledgeable, because nutrition education programmes had been offered over the years. Nutrition education for staff was emphasized as a measure to prevent malnutrition. They stated that they were obligated to support and ensure continuing education, when it was needed.

'The caregivers are educated in nursing care, I know that nutrition education programmes have been provided'

If there was still lack of knowledge, the politicians and civil servants recommended more education addressing this area. They recommended education based on a holistic approach to the patient, which would allow staff to better identify nutritional problems and take appropriate actions to remedy them.

'The nutritional courses need to go deep, to see what is behind things, to see the person'

#### Govern at a distance

The politicians and civil servants mentioned that, within their area of responsibility, they govern at a distance. They reported that when signals of nutritional problems appeared at their level in the healthcare system, on their agenda, they acted by turning to the local management level. The role of local management was rarely mentioned, and this also applies to regular follow-ups with local management and staff.

'Nutrition is not an uncommon issue on our agenda, when it appears the matter has to be dealt with until it is resolved, with the management'

### Moral perception

#### Discomfort

The politicians and civil servants mentioned that caregivers in elder care often worked in a hurried, task-oriented manner that did not address the individual patient's needs. This situation led to uneaten food among vulnerable patients, according to the participants. They found situations such as these distressing.

Everything had to go so fast, they (the caregivers) whip in, the food is uneaten, and they don't see it, it's not right'

#### Trust and distrust

The politicians and civil servants expressed feelings of trust and distrust in relation to the staff. They believed staff members were competent in addressing nutritional issues owing to their role and education as providers of nursing care. At the same time, they expressed their distrust in that they wondered whether caregivers adapted meals to each patient's needs, and they felt this situation was problematic. Nothing was mentioned about such aspects in relation to local management.

'I don't know if they haven't got time or if they haven't taken time for the meals, the patient is ill. In my job I must trust the caregivers, but now I feel uncertain'.

Distrust was experienced when signs of malnutrition among elderly patients reappeared on their agenda. The participants thought that, because educational programmes had been provided, the staff should be competent to deal with various nutritional situations, i.e., they should notice patients who are not eating or who have lost weight.

If the caregivers who serve food every day don't notice that the elderly are not eating and are losing weight, then they must be blind or something'.

## Discussion

Malnutrition was stressed as an important dimension of the quality of elder care. The roles assumed were used as a basis for assignment of ethical responsibility. Caregivers were considered to be primarily responsible for ensuring patients' nutritional status and were expected to be competent. The politicians' and civil servants' role and overall ethical responsibility for the quality of care and costs was experienced as difficult, and associated with increasing care needs versus pressures to cut costs. Reversing malnutrition among older, ill patients was believed to be difficult. Trust and distrust were revealed, and the distrust reflected doubts as to whether these issues could be addressed appropriately in the healthcare system. Governing at a distance meant having trust in the staff, on the one hand, and discomfort and distrust when confronted with reports of malnutrition, on the other. Distrust was directed at caregivers, because despite the fact that nutrition education programmes had been provided, problems reappeared. Physicians' responsibility was required, though the accountability of local management and the staffing situations were hardly mentioned. Nutritional problems were addressed when they appeared on the respondents' agenda.

In our study, malnutrition was considered an important issue with regard to quality of care, but the politicians and civil servants often experienced their role and overall ethical responsibility as difficult to live up to. This was mentioned particularly in regard to increasing care needs and attempts to meet these needs within limited budgets. There is a disparity between welfare state ambitions and the available resources, according to Bakken and co-workers [27]. There is an inherent ambiguity in care even when nursing care is planned, made more effective and organized. This ambiguity will persist, and there is a need for a public debate on the needs of care and the exclusions that are allowed [28].

In our study, the participants spoke of quality of care and malnutrition in relation to the fact that many patients in elder care are old and ill, which was seen as the major cause of malnutrition. However, if this perspective becomes too predominant, it can lead to narrow-mindedness and to other important perspectives being neglected. When this happens, care providers may feel that there is little meaning to providing care. Moreover, this perspective could also lead to politicians and civil servants not assuming the ethical responsibility for following up issues of malnutrition among vulnerable persons in elder care.

The politicians and civil servants emphasized that it was caregivers who had the primary responsibility for dealing with issues of malnutrition. Yet such a focus on caregivers alone must be considered too limited. It would seem to be necessary for those with top positions in the healthcare system to also become familiar with the area of concern [29], its complexity and the often ethically challenging caring situations faced by caregivers every day [11].

The participants expected caregivers to be competent and to address nutritional issues appropriately. Education programmes had been provided, according to the politicians and civil servants, and if caregivers still lacked knowledge, more education was recommended. According to Kayser-Jones [30], training and supervision of staff are often paramount to resolving problems in nursing homes. Education is regarded as a cost-effective way to increase caregivers' knowledge of nutrition and to ensure adequate food intake among patients, but few studies have actually evaluated the results of such interventions [31]. Watson and Green [32] called for standardized research interventions in the area of food provision to persons with dementia. Further research is needed concerning nutrition education programmes in elder care; the content, the form and the implementation.

Christensson and co-workers [33] reported positive attitudes towards nutritional nursing care after an education programme, especially concerning the importance of food. However, a smaller proportion of staff members demonstrated a positive attitude regarding patients' self-ability. In contrast, an education programme in integrity-promoting care [34] was beneficial, in that it contributed to a positive atmosphere during mealtimes and facilitated interaction, which indirectly led to weight gain and increased intellectual functioning among patients on an intervention ward. Thus, the dimension of human relationships seems to be important in nutrition education programmes [34]. The high rate of turn over among the staff in elder care [35] is probably an aspect that opposes the nutritional education programmes. Local management is responsible for carrying out continuing education in a healthcare system. In our study, the role of local management was only rarely mentioned.

Moral perceptions such as discomfort were felt by the politicians and civil servants when the nutritional routines were not conducive to proper nutrition. According to Sidenvall and co-workers [36] mealtimes in institutions were carried out in such a way that care was not suited to older adults' needs. Mattsson-Sydner and Fjellström [37] stressed that mealtime situations are often shaped by individuals' living arrangements and not by individuals' needs, and a limited amount time is allotted to meals. Thus, when dealing with nutritional issues many aspects have to be considered - every thing from well-functioning relationships, to adequate staffing and good routines.

In our study, the participants mentioned how they governed at a distance, which meant that they had to trust the staff. However, feelings of distrust were expressed regarding caregivers' failure to meet patients' nutritional needs. The politicians and civil servants placed the primary responsibility for nutritional issues on caregivers and focused less on other aspects of the healthcare system. In contrast, Sorlie and co-workers [11] reported on enrolled nurses who placed the responsibility for their work situation, especially lack of resources, on authorities, administrators and the healthcare system.

Increased physician responsibility was requested by the politicians and civil servants owing to the complexities associated with multiple illnesses in elder care. Cederholm [38] stated that nutritional issues must be dealt with as seriously as other medical problems. In Sweden, the limited involvement of physicians may mirror the lack of shared responsibility in elder care between two healthcare systems: the local municipalities and the county councils. It may also reflect physicians' interest in nutrition.

The participants made few comments regarding their own working routines, role and ethical responsibility in the problem area. This is understandable, given that they placed the primary responsibility on caregivers. This probably mirrors politicians' and civil servants' expectations with regard to the system, which assumes that those who are delegated a task, will perform it correctly. On the other hand, they felt discomfort when confronted with examples of poor nutritional care, which indicates that they experienced failure in their ethical responsibility because the quality of care was at risk. Local management's responsibility for nutritional issues was only briefly mentioned, making them almost invisible. This may be because the politicians and civil servants see this as a medical issue, but it is nevertheless curious that local management were mentioned so rarely, as nutritional issues are associated with their area of responsibility. This is confirmed by other studies showing that local management leaders must take more responsibility for nutritional issues in elder care [21,22].

One of the most basic elements of elder care is ensuring an adequate diet and proper nutrition. Not fulfilling this obligation can be seen as an ethical question on the individual as well as system level. Not only individuals but also healthcare systems can be referred to as unethical if they lack structure and prerequisites for ethical defensible care [13]. The different levels of the system are intertwined, and there seem to be gaps in the dialogue between them, which may lead to "finger pointing". Not having dialogues may negatively affect the sense of responsibility of those involved and lead to a sense of powerlessness at all levels [24].

## Methodological consideration

Interviews have been found appropriate for this study since the aim was to grasp an extended understanding of politicians' and civil servants' experiences and reasoning about malnutrition in elder care related to ethical responsibility. Persons holding high positions belong to a limited group in a health care system. Therefore, they were recruited from two counties to decrease the risk of participant recognition. However, this study has limitations. The present interviews were performed shortly after another interview session. This could have lead to participants' tiredness. The pause in-between was meant to counteract such feelings. Another threat could be related to the participants' experiences and skills in interview situations, where they often are given general answers instead revealing deeper reasoning, which was desired here. During these interviews, the politicians and civil servants were able to talk about what was important to them. In interviews that focus on difficulties, an indirect idea of what is 'good' may arise through descriptions of what is lacking or what is at stake [39]. Eighteen participants were interviewed, and the risk of data redundancy was considered before the start of the study. The phenomenological hermeneutic method seemed to be useful here, as the aim was to illuminate lived experiences [25]. However, the naïve reading of the text will determine whether it is possible to perform such an analysis. If a text consists of more descriptions than lived experiences, then a latent content analysis is preferable. Our text proved to be rich, highly detailed and consisted of many thoughts and feelings, which made the phenomenological hermeneutic method suitable. This method enables interpretation of lived experiences and can provide a basis for reflection on the area of malnutrition, as confronted by politicians and civil servants in elder care. Quotes are presented and all the authors took part in the analyses in order to increase the credibility of the study. The present results cannot be generalized, but are credible if persons with similar experiences recognize and can relate to the descriptions or interpretations [40]; if this is the case, the results may be applied to similar situations [26].

## Conclusion

The present study contributes new knowledge on the issue of malnutrition in relation to ethical responsibility, as illuminated by persons who had top positions in elder care and who were accountable for the budget and quality of care. The politicians and civil servants felt discomfort when confronted with examples of poor nutritional care. This indicates that they experienced failure in their ethical responsibility because the quality of nutritional care was at risk. The ethical responsibility for upholding nutritional quality in elder care should be more equally distributed between staff, local management and persons holding top positions. Dialogues promoting shared understanding, not only of good nutritional standards, but also of the ethically sound prerequisites that must be in place.

## Competing interests

The authors declare that they have no competing interests.

## Authors' contributions

AGM participated in the design of the study, carried out the interviews, participated in the analysis, completed and approved of the final manuscript. MK and VS participated in the design of the study, read the interviews, participated in the analysis, helped to complete and approved of the final manuscript.

## Pre-publication history

The pre-publication history for this paper can be accessed here:

http://www.biomedcentral.com/1472-6939/11/11/prepub
